# Therapeutic Potential of *Gynostemma pentaphyllum* Extract for Hair Health Enhancement: A Randomized, Double-Blind, Placebo-Controlled Clinical Trial

**DOI:** 10.3390/nu17050767

**Published:** 2025-02-21

**Authors:** Jihyun Lee, Yongxun Jin, Xinrui Zhang, Myoungrae Kim, Ayoung Koh, Shuyi Zhou, Changhyun Lee, Minji Seo, Shinjae Kim, Suye Jo, Youngjoo Kim, Seri Kwon, Kyuhan Kim, Chanyeong Heo

**Affiliations:** 1 Easy Hydrogen Corporation, Jeju 63196, Republic of Korea; easyhydrogen@gmail.com (J.L.); xincaide1@naver.com (S.K.); 2 Department of Plastic and Reconstructive Surgery, College of Medicine, Seoul National University, Seoul 03080, Republic of Korea; jinyongxun789@snu.ac.kr (Y.J.); zhangxinrui@snu.ac.kr (X.Z.); shuyi_chow@snu.ac.kr (S.Z.); 3 Department of Plastic and Reconstructive Surgery, Seoul National University Bundang Hospital, Seongnam 13620, Republic of Korea; 4 Korean Skin Research Center, Seongnam 13558, Republic of Korea; mrkim@koreansrc.com (M.K.); mjseo@koreansrc.com (M.S.); sycho@koreansrc.com (S.J.); srkwon@koreansrc.com (S.K.); 5 Department of Biological Engineering, Graduate School of Konkuk University, Seoul 05029, Republic of Korea; aykoh@koreansrc.com; 6 Department of Urology, College of Medicine, Jeju National University, Jeju 63243, Republic of Korea; kurology@naver.com; 7 Department of Dermatology, Veterans Health Service Medical Center, Seoul 05368, Republic of Korea

**Keywords:** *Gynostemma pentaphyllum*, natural therapeutics, hair health, follicular regeneration, safety, antioxidant effects

## Abstract

**Background:** Hair health critically influences both aesthetic appearance and psychological well-being. Existing treatments often show limited efficacy and may cause side effects. *Gynostemma pentaphyllum* (GP), known for its antioxidant and anti-inflammatory properties, has emerged as a promising botanical agent, although clinical evidence regarding its hair health benefits remains limited. **Purpose:** This study aimed to evaluate the efficacy and safety of GP extract in improving hair parameters through a randomized controlled trial. **Methods:** This randomized, double-blind, placebo-controlled trial involved 100 eligible adults aged 19–60 years who were randomly allocated to either the GP or placebo group. Participants consumed 340 mL/day of the test product for 24 weeks. The primary outcomes included hair elasticity, density, diameter, glossiness, and subjective satisfaction. Safety was evaluated through laboratory tests and adverse event monitoring. **Results:** After 24 weeks, the GP group showed a threefold increase in hair elasticity and density and a fourfold increase in hair diameter compared to the placebo group. The subjective satisfaction scores corroborated these findings: the GP users reported better outcomes in terms of reducing hair damage and dryness. No significant differences in hair glossiness were observed based on the instrumental and visual assessments (*p* > 0.05). The safety evaluations revealed no severe adverse events. All the safety evaluation metrics demonstrated no significant abnormalities. **Conclusions:** This study provides compelling evidence of the efficacy of GP extract in enhancing hair health, demonstrating both significant functional improvements and an excellent safety profile. These findings substantiate its potential as a promising functional food ingredient for comprehensive hair care interventions.

## 1. Introduction

Hair health serves as a complex physiological indicator integrating multiple parameters, including the follicular density, mechanical properties of the hair shaft, surface reflectivity, and dimensional characteristics. The integrity of these attributes is vulnerable to a spectrum of deleterious factors, encompassing tobacco exposure, nutritional deficiencies, psychological stressors, pharmacological interventions, pathological conditions, chemical agents, inflammatory cascades, and metabolic dysregulation [1,2]. These factors often lead to reduced follicular density, weakened hair structure, decreased shine, and compromised elasticity, contributing to noticeable hair deterioration. The implications of compromised hair health transcend purely dermatological and aesthetic considerations, imposing substantial psychosocial consequences that can significantly impair self-esteem and overall quality of life [3]. Consequently, improving hair health has become a critical societal issue, necessitating multidisciplinary attention and innovative solutions.

The fundamental approach to enhancing hair health centers on the optimization of follicular stem cell functionality, amplification of the regenerative potential, and extension of the anagen phase while maintaining follicular integrity against environmental and physiological insults. Contemporary therapeutic strategies primarily encompass nutritional interventions with essential micronutrients, behavioral modifications, and targeted pharmacological approaches [4]. Minoxidil exemplifies this approach through its vasodilatory action, which augments follicular perfusion and optimizes the perifollicular microenvironment, resulting in enhanced follicular density and structural integrity. Novel therapeutic modalities, including immunomodulatory agents such as Janus kinase (JAK) inhibitors and regenerative interventions utilizing platelet-rich plasma (PRP) and dermal sheath cup cell transplantation, have demonstrated efficacy in terms of follicular regeneration and density enhancement [5,6,7,8]. However, these interventions are frequently accompanied by adverse effects, including endocrine disruption, cutaneous irritation, sexual dysfunction, aberrant hair growth, and cephalgia, while their therapeutic benefits often prove temporary [9]. Moreover, the longitudinal safety profile of systemic interventions remains incompletely characterized. Despite these advances in therapy, safer and more effective long-term interventions are still needed.

Natural therapeutic agents, particularly botanically derived bioactive compounds, have gained increasing attention for their enhanced safety profiles and long-term benefits in promoting hair health. Among them, *Curcuma longa* plays a crucial role in scalp health maintenance by exerting anti-inflammatory and antioxidant effects, while also modulating hormonal activity to support the follicular balance. Likewise, *Camellia sinensis* (green tea extract), rich in epigallocatechin gallate, promotes hair follicle proliferation and dermal papilla cell protection, contributing to stronger, healthier hair. *Gynostemma pentaphyllum* (GP), a traditional medicinal herb containing a diverse array of pharmacologically active constituents, including gypenosides, flavonoid compounds, and complex polysaccharides, has demonstrated significant therapeutic utility in traditional Chinese medicine for managing various chronic pathologies, including hypertension, dyslipidemia, and glucose metabolism disorders [10,11]. It demonstrates remarkable antioxidant and anti-inflammatory effects, positioning it as a promising intervention for addressing hair health [12,13]. However, its potential to promote hair growth has been less extensively studied. Our preclinical investigation revealed that GP extract demonstrates significant efficacy in promoting follicular regeneration through dual mechanisms: stimulation of dermal papilla cellular activity and activation of the Wnt/β-catenin signaling cascade, a fundamental regulatory pathway in follicular regenerative processes [14]. This mechanistic action results in the prolongation of the anagen phase, with consequent enhancement of the follicular density, augmentation of the hair shaft diameter, and optimization of the overall hair structural parameters. The observed trichogenic effects demonstrate comparable efficacy to minoxidil, the current therapeutic standard. Complementary preclinical investigations by Liu et al. elucidated that GP extract significantly enhances hair’s morphophysiology through activation of these molecular pathways while simultaneously suppressing growth-inhibitory gene expression [15]. The documented effects encompass prolongation of the anagen phase duration, enhancement of the follicular dimensions, increased follicular density, and amelioration of hair pigmentation through upregulated melanogenesis, collectively resulting in improved hair optical properties. These findings elucidate the molecular mechanisms underlying *Gynostemma pentaphyllum*’s trichogenic properties and substantiate its potential as a therapeutic agent for hair health optimization.

This study hypothesized that GP extract contributes to hair health enhancement by promoting follicular regeneration and improving key hair characteristics, including the elasticity, density, diameter, and glossiness, while maintaining a favorable safety profile without significant adverse effects. To systematically evaluate these effects, a methodologically rigorous 24-week randomized, double-blind, placebo-controlled clinical trial was conducted. This study aimed to assess the therapeutic efficacy of GP extract in improving hair parameters relative to a placebo while also examining its safety profile, including the biochemical markers, hormone levels, and potential adverse events [16]. Through addressing fundamental knowledge gaps in therapeutic hair health management, this investigation aimed to establish evidence-based validation of GP extract’s therapeutic potential, thereby contributing to the development of evidence-based interventions for hair health optimization.

## 2. Materials and Methods

### 2.1. Trial Product and Trial Design

The hydrodistillate of GP was developed by Easy Hydrogen Corporation (Jeju City, Korea) and prepared by Chunjieh Cooperation (Jeju City, Korea) in accordance with the Good Clinical Practice (GCP) guidelines and methods described in the relevant literature [17]. First, 20 kg of *Gynostemma pentaphyllum* leaves was added to 1.2 tons of water in a pressurized concentrator with a pressure of 1.5 atmospheres and a temperature of 109 °C, then heated for 15 h. The vapor was collected, condensed, and sterilized at 95 °C for 1 h, then transferred to a raw material tank, sealed, and subsequently packaged into 340 mL test products. The heavy metals, including lead, cadmium, total arsenic, and total mercury, were all below 0.5 milligrams per kilogram, and the coliform bacteria tested negative. Previous in vitro and in vivo studies have demonstrated the significant promotive effects of this extract in relation to hair growth [14]. This study was approved by the Institutional Review Board (IRB) of the Korean Skin Research Center (approval number: HBSE-PFHA-23002) and strictly adhered to the ethical principles outlined in the Declaration of Helsinki.

This study was a 24-week randomized, double-blind, placebo-controlled trial that included 100 participants (50 in the GP group and 50 in the placebo group). Participants were recruited through public advertisements in local hospitals and offline platforms. Prior to the commencement of the trial, all the participants were provided with detailed information about this study’s objectives, its procedures, the expected benefits of the functional food, and the potential risks or adverse effects. The trial began only after obtaining voluntary written informed consent. Only those volunteers who signed the consent form underwent screening to ensure they met the inclusion criteria for this study.

Within four weeks of the screening visit, participants completed a baseline assessment that included the collection of demographic information, physical activity data assessed using the Global Physical Activity Questionnaire (GPAQ) to calculate the metabolic equivalent task (MET), dietary intake records, anthropometric measurements (height, weight, and body mass index, BMI), and information on alcohol consumption and smoking habits. Participants who met the inclusion and exclusion criteria were officially enrolled in the trial and began consuming the test product.

Participant allocation was conducted using block randomization to ensure a balanced group distribution. A computer-generated randomization sequence was implemented, with the allocation information rigorously concealed from both the investigators and the participants to maintain study blinding and minimize potential bias. For 24 weeks, participants in both groups consumed 340 mL of the designated test product prepared in advance by the research unit and uniformly distributed during follow-ups. The product was taken on an empty stomach before breakfast (between 6:00 and 8:00 a.m.), not with meals, and participants were instructed to avoid intense physical activity for 30 min to ensure consistent absorption. The GP group received GP extract and the control group was offered a placebo formulation carefully matched to the GP product. The placebo contained purified water with food-grade coloring and flavoring agents to match the GP product. This approach ensured visual, chromatic, and organoleptic equivalence between the GP and placebo products, thereby preserving this study’s double-blind design and preventing participant identification of the assigned group.

**Figure 1:**
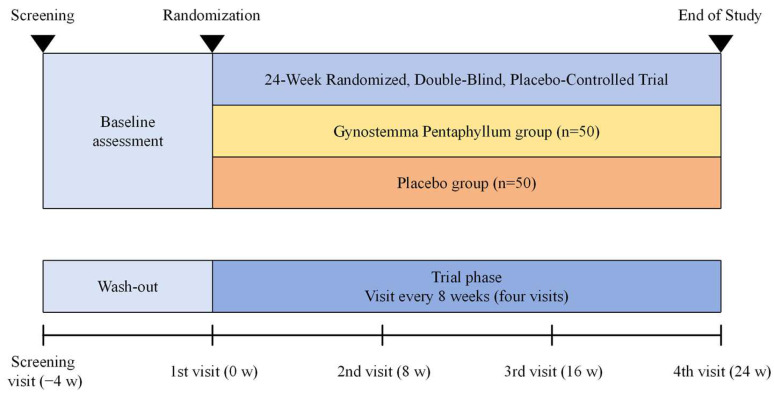
Schematic diagram of the trial visits.

Participants attended follow-ups every eight weeks for assessments. Figure 1 facilitates a more intuitive understanding of the visit process. These visits encompassed multiple critical activities, including executing the requisite tests, systematically collecting data, managing test product distribution and return to evaluate participant adherence, and implementing real-time monitoring of key evaluation parameters such as medication compliance, physical activity levels, and dietary intake. The meticulously structured follow-up protocol was explicitly designed to enhance this study’s scientific rigor, ensure data reliability, and proactively mitigate potential sources of methodological bias throughout the 24-week research investigation.

### 2.2. Inclusion Criteria

This study was conducted in South Korea from 25 April to 10 October 2025, spanning spring, summer, and autumn, with temperatures ranging from 10 °C to 35 °C. Eligible participants were healthy adults (19–60 years) with no history of hair-loss-related medical conditions. Eligible individuals had moderately damaged hair, as determined by visual assessment (glossiness score of 1–3) and a total hair damage score of 7–18 based on exposure to risk factors (Table 1). The scoring system accounted for chemical treatments (bleaching, perming, coloring), mechanical stress (combing, blow-drying, flat-ironing), protective care (conditioning, heat protection spray), and environmental exposure (sunlight). Participants were required to refrain from using specialized hair care products or undergoing hair treatments, maintain a consistent hairstyle, and avoid wearing headgear or sun-shading accessories throughout the study period. They had to fully understand the study objectives and requirements after receiving a comprehensive explanation, voluntarily consent to participate, and provide written informed consent. Additionally, participants had to commit to adhering to all the study-related precautions.

### 2.3. Exclusion Criteria

Individuals who had experienced severe acute kidney or heart disease, or other chronic conditions (such as hypertension or diabetes) that may affect the trial outcomes, within six months prior to screening; individuals with infectious skin diseases; individuals who had undergone hair-loss-related surgical treatments, such as hair transplantation or scalp reduction surgery; individuals who had taken oral dutasteride or finasteride within six months prior to screening; individuals who had applied topical hair growth agents or medications promoting hair growth within one month prior to screening; individuals who had used any of the following medications within one month prior to screening: steroids, cytotoxic agents, vasodilators, antihypertensive drugs, antiepileptic drugs, beta-blockers, bronchodilators, diuretics, spironolactone, cimetidine, diazoxide, cyclosporine, or ketoconazole; individuals who had applied topical corticosteroid preparations to the scalp within one month prior to screening; individuals diagnosed with hair loss conditions other than androgenic alopecia, such as alopecia areata, telogen effluvium, or diffuse alopecia, or with a family history of hair loss; individuals who had used products affecting hair growth within three months prior to screening or were currently using such products; individuals who met the World Health Organization (WHO) definition of heavy drinking: for men who consume more than 280 g of pure alcohol per week (equivalent to 20 standard drinks), or 40 g per day on average, and for women who consume more than 140 g per week (equivalent to 10 standard drinks), or 20 g per day on average; individuals requiring treatment for clinically significant acute or chronic cardiovascular, endocrine, immune, respiratory, hepatic, renal, urinary, neurological, psychiatric, musculoskeletal, inflammatory, hematologic–oncologic, or gastrointestinal diseases; individuals with a history of clinically significant allergic reactions to GP; individuals who had undergone antipsychotic drug therapy within three months prior to screening; individuals with a history of substance abuse or suspected substance abuse; individuals who had participated in other human trials within three months prior to screening; individuals with laboratory results meeting the following criteria: AST (aspartate aminotransferase) or ALT (alanine aminotransferase) greater than three times the upper limit of the reference range, or serum creatinine greater than 1.5 mg/dL; pregnant or breastfeeding women; women within six months postpartum; women of childbearing potential who did not agree to use appropriate contraceptive measures; and individuals deemed unsuitable for this study by the investigator based on test results or other reasons were excluded.

### 2.4. Withdrawal Criteria

Participants will be withdrawn from this study if any previously stated exclusion criteria or adverse effects are identified during the trial. In cases of serious adverse reactions, treatment will be discontinued based on a physician’s evaluation and appropriate standard medical care will be provided.

### 2.5. Evaluation Methods for Treatment Effectiveness

#### 2.5.1. Instrumental Evaluation

During the first and fourth visits, the same test site (1 square centimeter, marked for identification) underwent shaving. Measurements of the hair elasticity, glossiness, diameter, and density were then conducted on the shaved area.

Hair elasticity evaluation: Hair elasticity was measured using a universal testing machine (Advanced Materials Evaluation and Testing, Urbana, IL, USA). Three strands of hair were stretched at a constant pressure and speed, with the tension measured ten times in total. The results were recorded in gram-force (gf) units, and the average value was calculated for analysis.

Hair glossiness evaluation (instrumental): Hair glossiness was assessed using the Skin-Glossymeter GL 200 (C+K, Cologne, Germany). Each measurement was performed three times, and the average value was recorded in gloss units (GUs). The measurements were conducted during the first and fourth visits.

Hair diameter evaluation: Hair diameter was measured using a microscope (OLYMPUS, Tokyo, Japan) and ToupLite software 2.1.27501 (ToupTek, Hangzhou, China). Images were taken at a position 1 cm from the hair root for 10 strands of hair. The Image Pro^®^ Plus 10 software (Media Cybernetics, Rockville, MD, USA) was used to measure the diameter of the hair strands (μm), and the average value was calculated for analysis.

Total hair count per unit area: To evaluate the total number of hairs per unit area, the hair was trimmed from the marked area and scalp images were captured using the Folliscope 5.0 (Lead M, Seoul, Korea). During the first and fourth visits, the same test site (approximately 1 square centimeter, marked) was shaved and photographs were taken. The images were then analyzed to calculate the total hair count within a 1-square-centimeter area (number/cm^2^). A standardized marking method used with safe, non-toxic dye was applied with a fine-point micro-applicator at baseline and reinforced during follow-ups for consistency.

#### 2.5.2. Visual Evaluation

Visual assessments of hair glossiness and distribution were conducted independently by three experts before the intake of the test product and at weeks 0, 8, 16, and 24. The evaluations were scored based on standardized criteria.

Expert evaluation of hair distribution: Visual assessments were conducted under standardized conditions, including consistent posture, distance, and lighting for the test subjects. The hair distribution was evaluated using images captured at two angles: the frontal hairline at a 45° angle and the top of the head at a 90° angle. A 7-point scale was used for scoring: −3 indicated significant deterioration, −2 moderate deterioration, −1 slight deterioration, 0 no change, +1 slight improvement, +2 moderate improvement, and +3 significant improvement. The average score from the three experts was calculated for analysis.

Expert evaluation of hair glossiness (visual): Hair glossiness was assessed visually after shampooing and drying the hair under consistent lighting conditions. A five-point scale was used for scoring: 1 for almost no glossiness, 2 for slightly less glossiness, 3 for normal glossiness, 4 for slightly more glossiness, and 5 for very high glossiness. The average score from the three experts was calculated for analysis.

### 2.6. Satisfaction Score Evaluation

A survey consisting of seven questions was conducted to assess participants’ perceptions of their hair condition: 1. Do you feel your hair is damaged? 2. When combing your hair after drying, do you experience difficulty or resistance? 3. Do you feel your hair lacks glossiness? 4. Do you feel your hair is prone to breakage? 5. Do you feel your hair is dry? 6. Do you feel your hair lacks elasticity? 7. Do you feel your hair is rough? Each question was scored on a scale from 1 to 10, with lower scores indicating greater satisfaction.

### 2.7. Safety Assessment

Glomerular filtration rate (GFR): The GFR was estimated by measuring the blood creatinine concentration and applying the CKD-EPI calculation formula.

Safety assessment via interviews: During the treatment period, safety was monitored through telephone follow-ups and regular in-person visits, including interviews to assess subjective and objective symptoms as well as adverse reactions.

Laboratory tests to assess health status: Hematology: White blood cell count (WBC), red blood cell count (RBC), hemoglobin (Hb), hematocrit (Hct), and platelet count (PLT). Blood biochemistry: Alkaline phosphatase (ALP), gamma-glutamyl transferase (gamma-GT), aspartate aminotransferase (AST), alanine aminotransferase (ALT), total bilirubin, total protein, albumin, blood urea nitrogen (BUN), creatinine, total cholesterol, triglycerides, high-density lipoprotein cholesterol (HDL-C), low-density lipoprotein cholesterol (LDL-C), glucose, creatine kinase (CK), and lactate dehydrogenase (LDH). Urinalysis: Specific gravity, pH, WBC, nitrites, protein, glucose, ketones, urobilinogen, bilirubin, and occult blood. Pregnancy test: Urinary human chorionic gonadotropin (HCG).

Vital signs and physical examination: At each visit, participants’ vital signs, including the systolic and diastolic blood pressure and pulse rate, were measured.

Hormone testing (testosterone): The testosterone levels were measured at the screening visit (before treatment) and at the fourth visit (after 24 weeks of treatment).

### 2.8. Statistical Analysis

Data were analyzed using SAS software, version 2024.03, with statistical significance set at *p* < 0.05.

The efficacy evaluation involved analyzing the hair elasticity, glossiness, total hair count, hair diameter, and satisfaction scores using descriptive statistics, independent sample *t*-tests for the between-group differences, and paired *t*-tests for the within-group changes. The repeated measures variables (glossiness and expert evaluations) were analyzed with RM-ANOVA or linear mixed models, while ANCOVA was used to adjust for baseline imbalances. The safety evaluation included summarizing the adverse events by frequency and percentage, with chi-square or Fisher’s exact tests for the group comparisons. The laboratory test results and vital signs were analyzed descriptively, with independent sample *t*-tests for the between-group differences, paired *t*-tests for the within-group changes, and RM-ANOVA or linear mixed models for the repeated measures.

## 3. Results

### 3.1. Participant Sample and Characteristics

**Figure 2:**
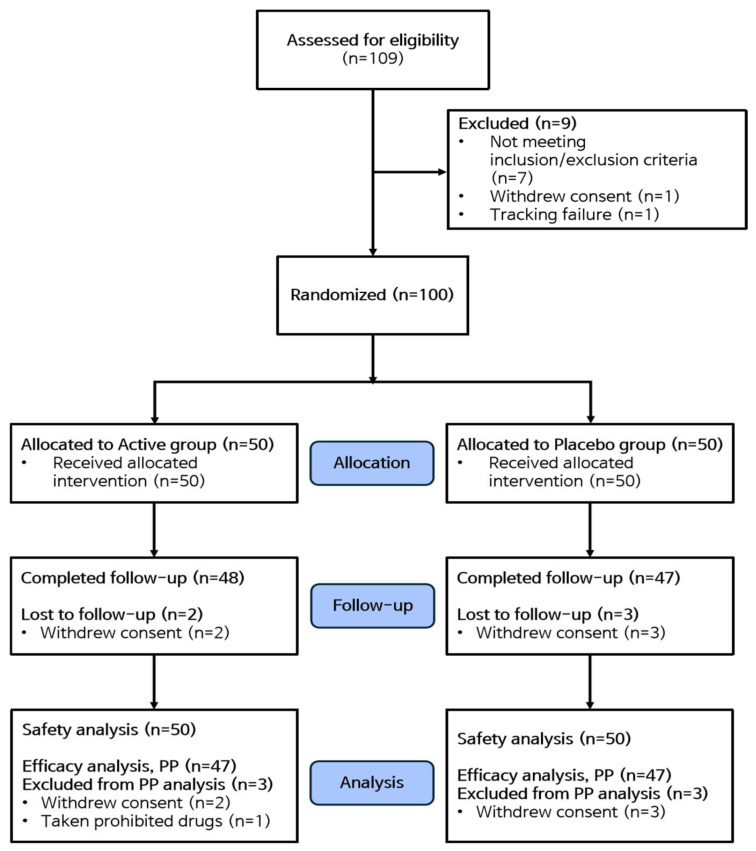
Schematic illustration of the trial protocol.

A total of 109 volunteers provided written informed consent and underwent screening, of whom 100 participants met the inclusion criteria and were enrolled in this study. During the trial, five participants withdrew consent—two from the GP group and three from the placebo group. Additionally, one participant from the GP group was excluded due to the use of prohibited concomitant medication. Ultimately, 94 participants completed the trial as per the protocol and were included in the efficacy analysis group. The safety analysis group included all 100 participants who consumed the trial product at least once (Figure 2).

Demographic information about the 100 study participants was analyzed, and the results are summarized in Table 2. Among the participants, there were 18 males (11 in the GP group and 7 in the placebo group) and 82 females (39 in the GP group and 43 in the placebo group). The average height was 163.96 ± 7.48 cm (166.26 ± 7.27 cm in the GP group and 161.66 ± 7.02 cm in the placebo group), and the average weight was 61.75 ± 10.21 kg (64.43 ± 11.02 kg in the GP group and 59.07 ± 8.63 kg in the placebo group). Both height and weight showed statistically significant differences between the groups (*p* = 0.002, *p* = 0.008), and the adjusted statistical results for height and weight are presented in Section 3. No significant differences were found between the groups for the other variables (*p* > 0.05).

### 3.2. Participant Medication Compliance

The compliance rate was calculated using the following formula: Compliance (%) = (Number of products actually consumed/Number of products expected to be consumed) × 100.

During the study period, the average number of products expected to be consumed per participant was 168.50 ± 2.42 bottles, while the average number of products actually consumed was 167.74 ± 4.23 bottles. The mean compliance rate was 98.74 ± 1.97%, with 98.79 ± 2.00% in the GP group and 98.69 ± 1.95% in the placebo group. There was no statistically significant difference in compliance between the two groups (*p* > 0.05) (Table 3).

### 3.3. Baseline Survey on Lifestyle

The physical activity levels were assessed using the GPAQ, with the T-MET (total metabolic equivalent of task) serving as the indicator. Higher T-MET values indicate greater physical activity levels. As shown in Table 4, comparisons of the T-MET values before and after 24 weeks of product intake revealed no statistically significant differences between the two groups (*p* > 0.05). Similarly, no statistically significant differences were observed between the two groups in terms of the body measurements, alcohol consumption, smoking habits, or dietary intake when comparing the values before and after 24 weeks of intake (*p* > 0.05).

### 3.4. Treatment Effectiveness

The evaluation of the treatment efficacy was conducted using the per protocol (PP) group, which included a total of 94 participants.

#### 3.4.1. Baseline of Efficacy Evaluation Indicators

The baseline measurements of the efficacy evaluation indicators, obtained during the screening and first visits, are summarized in Table 5.

The results showed a statistically significant difference in the total hair count per unit area between the two groups (*p* = 0.024). Consequently, statistical results adjusted for the baseline of the total hair count per unit area are provided. No statistically significant differences were observed between the groups for the other indicators (*p* > 0.05).

#### 3.4.2. Efficacy Evaluation (Instrumental)

Hair elasticity evaluation: Comparing the hair elasticity results before and after 24 weeks of intake, the change in the GP group was 58.14 ± 40.43 gf, while the placebo group showed a change of 19.92 ± 22.54 gf. The increase in the GP group was significantly greater than that in the placebo group, with a statistically significant difference between the groups (*p* < 0.0001). After adjusting for height and weight, the difference between the groups remained statistically significant (*p* < 0.0001) (Table 6).

Hair glossiness evaluation (instrumental): Insights can be drawn from Table 6, comparing the hair glossiness results (instrumental measurement) before and after 24 weeks of intake, where no statistically significant difference was observed between the two groups (*p* > 0.05).

Total hair count per unit area evaluation: Comparing the results of the total hair count per unit area before and after 24 weeks of intake, the change in the GP group was 14.04 ± 11.36 hairs/cm^2^, while the placebo group showed a change of 4.85 ± 8.60 hairs/cm^2^. The increase in the GP group was significantly greater than that in the placebo group, with a statistically significant difference between the groups (*p* < 0.0001). After adjusting for height and weight, the difference between the groups remained statistically significant (*p* < 0.0001).

**Figure 3:**
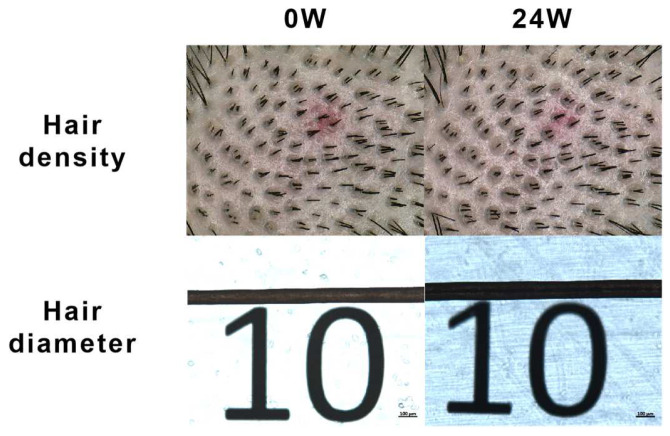
Photos of the same subject from the 1st and 4th visits (measured at the 1 cm diameter red tattoo mark).

Hair diameter evaluation: Comparing the hair diameter results before and after 24 weeks of intake, the change in the GP group was 17.49 ± 6.08 μm, while the placebo group showed a change of 3.63 ± 9.16 μm. The increase in the GP group was significantly greater than that in the placebo group, with a statistically significant difference between the groups (*p* < 0.0001). After adjusting for height and weight, the difference between the groups remained statistically significant (*p* < 0.0001). The differences before and after are more clearly and effectively illustrated in Figure 3.

#### 3.4.3. Efficacy Evaluation (Visual)

Expert evaluation of hair distribution: Comparing the evaluation results concerning the frontal hairline (45° angle) and the top of the head (90° angle) at different visits, no statistically significant differences were observed between the two groups (*p* > 0.05).

Expert evaluation of hair glossiness (visual): Comparing the visual evaluation results concerning the hair glossiness at different visits, no statistically significant differences were observed between the two groups (*p* > 0.05) (Table 7).

### 3.5. Satisfaction Score Evaluation

The survey results indicated that after 16 weeks of intake, the GP group showed significantly higher satisfaction compared to the placebo group for item 1 (hair damage), with scores of 4.91 ± 2.09 versus 5.77 ± 1.99 (*p* = 0.046), and for item 5 (hair dryness), with scores of 5.40 ± 1.81 versus 6.21 ± 1.81 (*p* = 0.033). After 24 weeks of intake, the GP group continued to demonstrate higher satisfaction for item 1 (hair damage), scoring 4.72 ± 1.87 compared to 5.51 ± 1.94 in the placebo group (*p* = 0.049). No statistically significant differences were observed between the two groups for the remaining items at any time point (*p* > 0.05) (Table 8).

### 3.6. Safety Assessment

The treatment efficacy evaluation was conducted using the safety group, which included a total of 94 participants.

#### 3.6.1. Glomerular Filtration Rate

A comparison of the glomerular filtration rate (GFR) results before and after 24 weeks of intake showed no statistically significant differences between the two groups (*p* > 0.05) (Table 9).

#### 3.6.2. Safety Assessment via Interviews

During the trial, among the 100 participants who consumed the test product, one mild adverse reaction occurred in the GP group, while no severe adverse reactions were reported. The incidence rate of adverse reactions showed no statistically significant difference between the two groups (*p* > 0.05) (Table 10).

#### 3.6.3. Laboratory Test Indicators

The laboratory test results, including the hematology, blood biochemistry, and urinalysis, indicated statistically significant differences between the two groups in terms of the total bilirubin (*p* = 0.016), glucose (*p* = 0.019), and high-density lipoprotein cholesterol (HDL-C) (*p* = 0.017). However, these changes were within the reference range and deemed clinically insignificant. No statistically significant differences were observed between the groups for the other parameters (*p* > 0.05) (Table S1).

#### 3.6.4. Vital Signs and Physical Examination

A comparison of the vital sign results across all the follow-up periods revealed no statistically significant differences between the two groups for any of the vital sign indicators (*p* > 0.05) (Table 11).

#### 3.6.5. Hormone Testing (Testosterone)

A comparison of the hormone testing results before and after 24 weeks of intake showed no statistically significant differences between the two groups (*p* > 0.05) (Table 12).

## 4. Discussion

### 4.1. Efficacy Evaluation

This 24-week randomized controlled trial investigated the therapeutic potential of GP extract in enhancing hair health parameters. This study revealed statistically significant improvements in the GP group compared to the placebo group, demonstrating a 14 hairs/cm^2^ increase in hair density, a 58 gf enhancement in hair elasticity, and a 17 μm expansion in hair shaft diameter. These findings suggest GP extract’s promising capability to stimulate hair growth and improve structural hair characteristics. The patient-reported outcomes corroborated the objective measurements, with participants in the GP group reporting substantially reduced hair damage and decreased dryness. Notably, the instrumental and expert assessments found no significant differences in hair glossiness between the GP and placebo groups, potentially due to methodological limitations such as the restricted sample size, insufficient intervention duration, or limited sensitivity of the glossiness evaluation protocol. Comprehensive evaluations of the frontal hairline and scalp vertex did not reveal statistically significant morphological changes, indicating a need for more precise participant selection criteria and refined research methodologies. Future research should extend the study duration, refine the assessment methods, and include participants with more severe hair health issues.

The efficacy evaluation aligns with our previous molecular studies [14,18], demonstrating that damulin B, as one of GP extract’s bioactive compounds, activates the Akt pathway and inhibits GSK3β, significantly upregulating the vascular endothelial growth factor (VEGF), keratinocyte growth factor (KGF), and insulin-like growth factor-1 (IGF-1). These molecular mechanisms enhance vascularization and microcirculation in hair follicles, supporting follicular proliferation and differentiation.

This study also supports the hypothesis that GP extract improves hair health through multiple molecular mechanisms. These include anti-inflammatory and antioxidant actions, where the saponins, flavonoids, and polysaccharides in GP extract reduce free radicals and inflammatory markers such as IL-6 and TNF-α, alleviating chronic inflammation and oxidative stress around hair follicles and extending the follicular growth phase [12]. Furthermore, GP extract optimizes mitochondrial function by modulating the AMPK/SIRT1 signaling pathway, restoring autophagy flow, and enhancing the mitochondrial oxidative phosphorylation efficiency, which reduces apoptosis and boosts energy metabolism [19,20]. In addition to these mechanisms, the anti-anxiety and anti-fatigue effects of GP extract help alleviate psychological stress, indirectly promoting hair growth [21]. Its prebiotic effects also balance the gut microbiota, reducing systemic inflammation and oxidative stress, thereby providing comprehensive support for hair follicle function [22,23]. These mechanisms highlight GP extract’s potential for improving hair health and support its role as a therapeutic agent [16,24].

Several plant-based formulations have demonstrated efficacy in enhancing hair growth through antioxidant, anti-inflammatory, and hormonal modulation mechanisms. A pumpkin seed oil intervention significantly increased the hair density and shaft thickness over 24 weeks, likely due to follicle stimulation and anti-inflammatory effects [25]. Similarly, a 24-week trial involving green tea and *Sophora flavescens* extract attributed improvements in the hair density and thickness to epigallocatechin gallate-mediated dermal papilla cell proliferation [26]. Consistent with these findings, GP extract, rich in polyphenolic antioxidants, may exert similar follicular regeneration effects, supporting its potential as a natural therapeutic agent for hair health.

### 4.2. Safety Assessment

The safety profile of GP extract was exceptionally robust in this study, with no reported serious adverse events. Unlike conventional hair loss medications such as minoxidil and finasteride, GP extract avoided local dermatological complications like skin irritation and dandruff, as well as systemic complications, including hypotension and cardiac irregularities [27]. Comprehensive laboratory assessments—encompassing hematological parameters, blood biochemistry, urinalysis, renal function evaluation, and hormonal panels—revealed no clinically significant abnormalities. The testosterone levels remained stable throughout the treatment, indicating a minimized risk of hormone-related side effects. These include sexual dysfunction, gynecomastia, and depression, which are frequently associated with long-term use.

Consistent with previous research on natural botanical extracts like saw palmetto, pumpkin seed oil, and green tea, GP extract exhibited outstanding clinical safety across trials spanning two to six months [28,29,30]. While minimal adverse reactions were observed (including occasional mild constipation, slight scalp sensitivity, or transient gastrointestinal discomfort), their frequency and intensity were substantially lower compared to traditional pharmacological interventions. The extract’s remarkable safety and tolerability profile position it as an optimal therapeutic agent, particularly for patients seeking gentler, long-term treatment strategies with minimal risk of adverse interactions or systemic complications.

### 4.3. Limitations and Future Research Directions

While this investigation provides compelling evidence supporting GP extract’s potential in terms of hair health restoration and corroborates previous cellular and preclinical research, several methodological limitations warrant careful consideration. This study’s participant cohort exhibited restricted demographic diversity, with potential constraints in age, ethnicity, and gender representation. The 24-week intervention period precluded comprehensive evaluation of the long-term therapeutic outcomes, and the relatively constrained sample size may have compromised the statistical generalizability. Although initial molecular mechanistic insights were obtained, further research is necessary to elucidate the full spectrum of GP extract’s biological interactions.

Future research should aim to validate the efficacy of GP extract in relation to different types of hair loss, such as androgenetic alopecia and alopecia areata. Exploring the potential synergistic interactions with established treatments like minoxidil and complementary antioxidant interventions could provide nuanced therapeutic insights. Expanding the research scope through larger, multicenter clinical trials would significantly enhance the findings’ representativeness and statistical robustness. Additionally, the 24-week intervention period was relatively short, limiting the ability to comprehensively assess the long-term therapeutic effects of GP extract. A longer follow-up period in future studies would be essential to evaluate the sustained efficacy and safety of GP extract over an extended duration. Moreover, comprehensive investigations into the individual bioactive constituents of GP extract and strategic formulation optimization are critical to maximizing its therapeutic potential in hair health management and related clinical applications.

## 5. Conclusions

This 24-week randomized, double-blind, placebo-controlled study comprehensively assessed the efficacy and safety of GP extract for hair health improvement. The findings substantiated GP extract’s potential as a functional food, not only offering an innovative approach to hair care but also providing a safer alternative and a scientific basis for the treatment of hair loss and related conditions.

## Figures and Tables

**Table 1:** Hair damage risk scoring table.

Action	More than 1 Time/6 Weeks	1 Time/6 Weeks~3 Months	Less than 1 Time/3 Months
Bleaching	+10 points	+5 points	+1 point
Perming	+10 points	+5 points	+1 point
Coloring	+10 points	+5 points	+1 point
Hair Cut	−3 points	−2 points	−1 point
Flat Iron	+5 points	+3 points	+1 point
Blow Dry	+3 points	+2 points	+1 point
Combing/Brushing	+3 points	+2 points	+1 point
Washing	+3 points	+2 points	+1 point
Deep Conditioning Treatment	−5 points	−3 points	−1 point
Rinse-Off Conditioner	−3 points	−2 points	−1 point
Heat Protection Spray	−3 points	−2 points	−1 point
Sun Exposure	+5 points	+3 points	+1 point

**Table 2:** Demographic information.

	GP Group (*n* = 50)	Placebo Group (*n* = 50)	Total (*n* = 100)	*p*-Value ^(1)^
Sex (M/F)	11/39	7/43	18/82	0.2982
Age (years)	40.42 ± 8.97	39.32 ± 10.12	39.87 ± 9.53	0.567
Height (cm)	166.26 ± 7.27	161.66 ± 7.02	163.96 ± 7.48	0.002 **
Weight (kg)	64.43 ± 11.02	59.07 ± 8.63	61.75 ± 10.21	0.008 **
BMI (body mass index) (kg/m^2^)	23.25 ± 3.19	22.55 ± 2.44	22.90 ± 2.85	0.221
SBP (systolic blood pressure) (mmHg)	115.76 ± 14.18	112.80 ± 13.26	114.28 ± 13.74	0.284
DBP (diastolic blood pressure) (mmHg)	80.84 ± 9.12	80.66 ± 8.31	80.75 ± 8.68	0.918
Pulse (times/min)	72.24 ± 10.04	74.54 ± 10.93	73.39 ± 10.50	0.276
Alcohol (*n*, %)	15 (30.00)	15 (30.00)	30 (30.00)	1.000 ^(2)^
Alcohol (g/week)	11.02 ± 19.20	15.22 ± 34.33	13.12 ± 27.75	0.453
Smoking (*n*, %)	2 (4.00)	6 (12.00)	8 (8.00)	0.269 ^(3)^
Pieces (cigarettes/week)	0.60 ± 3.14	1.36 ± 4.09	0.98 ± 3.65	0.300

Values are presented as the mean ± SD or number (%); ^(1)^ analyzed by independent *t*-test between the groups; ^(2)^ analyzed by chi-square test between the groups; ^(3)^ analyzed by Fisher’s exact test between the groups; ** *p* < 0.01.

**Table 3:** Participant medication compliance.

	GP Group (*n* = 47)	Placebo Group (*n* = 47)	Total (*n* = 94)	*p*-Value ^(1)^
Prescriptions (should be taken) (bottle)	168.38 ± 1.95	168.62 ± 2.43	168.50 ± 2.42	0.642
Total intakes (actually taken) (bottle)	167.74 ± 4.41	167.74 ± 4.10	167.74 ± 4.23	1.000
Compliance (%)	98.79 ± 2.00	98.69 ± 1.95	98.74 ± 1.97	0.808

Values are presented as the mean ± SD; ^(1)^ analyzed by independent *t*-test between the groups.

**Table 4:** Baseline survey on lifestyle.

	GP Group (*n* = 47)				Placebo Group (*n* = 47)				*p*-Value ^(2)^
	Baseline	24 Week	Change Value	*p*-Value ^(1)^	Baseline	24 Week	Change Value	*p*-Value ^(1)^	
Alcohol Consumption and Smoking Habits									
Alcohol (g/week)	9.83 ± 18.41	9.62 ± 17.87	−0.21 ± 6.75	0.830	15.77 ± 35.28	20.17 ± 40.38	4.40 ± 22.98	0.195	0.192
Pieces (cigarettes/week)	0.43 ± 2.92	0.64 ± 3.23	0.21 ± 1.46	0.323	1.23 ± 4.02	0.81 ± 2.89	−0.43 ± 2.92	0.323	0.184
Physical Activity									
T-MET (min/week)	2713.62 ± 4016.06	2211.49 ± 2714.64	−502.13 ± 3297.76	0.302	2233.19 ± 2311.83	2064.68 ± 1677.34	−168.51 ± 1641.78	0.485	0.537
Body Measurement Survey									
Weight (kg)	63.98 ± 11.17	64.07 ± 12.08	0.09 ± 1.91	0.744	58.68 ± 8.29	58.26 ± 8.56	−0.42 ± 2.09	0.174	0.218
BMI (kg/m^2^)	23.24 ± 3.24	23.24 ± 3.47	0.06 ± 0.25	0.967	22.50 ± 2.35	22.32 ± 2.37	−0.17 ± 0.80	0.146	0.256
Dietary Intake Survey									
Calories (kcal)	1420.67 ± 759.03	1439.26 ± 640.95	18.59 ± 749.95	0.866	1313.56 ± 542.91	1301.10 ± 479.74	−12.46 ± 674.13	0.900	0.833
Carbohydrates (g)	180.27 ± 74.04	198.15 ± 87.79	17.87 ± 89.35	0.177	176.23 ± 82.64	183.81 ± 73.01	7.58 ± 97.42	0.596	0.595
Fats (g)	52.21 ± 43.81	45.82 ± 28.13	−6.39 ± 39.83	0.277	42.91 ± 27.03	39.69 ± 21.64	−3.22 ± 30.10	0.467	0.644
Protein (g)	59.90 ± 39.49	59.12 ± 29.47	−0.78 ± 37.55	0.888	55.08 ± 29.62	52.61 ± 22.08	−2.48 ± 35.43	0.634	0.822
Dietary fiber (g)	12.96 ± 6.55	13.70 ± 6.73	0.74 ± 8.07	0.530	12.01 ± 5.58	12.44 ± 5.49	0.43 ± 6.30	0.640	0.835

Values are presented as the mean ± SD; ^(1)^ analyzed by paired *t*-test between baseline and 24 weeks within each group; ^(2)^ analyzed by independent *t*-test for change value between the groups.

**Table 5:** Baseline for the efficacy evaluation indicators.

		GP Group (*n* = 47)	Placebo Group (*n* = 47)	Total (*n* = 94)	*p*-Value ^(1)^
Hair Elasticity (gf)		217.02 ± 41.27	204.91 ± 44.54	210.97 ± 43.14	0.175
Hair Gloss	Visual Inspection (score)	1.53 ± 0.55	1.55 ± 0.65	1.54 ± 0.60	0.864
	Instrumental (GU)	3.11 ± 0.42	3.11 ± 0.42	3.11 ± 0.42	0.953
Total Hair Count Per Unit Area (number/cm^2^)		140.40 ± 27.31	152.98 ± 25.93	146.69 ± 27.23	0.024 *
Hair Diameter (μm)		90.84 ± 8.58	88.31 ± 10.53	89.58 ± 9.64	0.205
GFR [60 ≤ mL/min/1.73 m^2^]		107.77 ± 10.88	110.49 ± 10.65	109.13 ± 10.80	0.223

Values are presented as the mean ± SD; ^(1)^ analyzed by independent *t*-test between the groups; * *p* < 0.05.

**Table 6:** Hair assessment (instrumental).

	GP Group (*n* = 47)				Placebo Group (*n* = 47)				*p*-Value ^(2)^	Adj, *p*-Value ^(3)^
	Baseline	24 Week	Change Value	*p*-Value ^(1)^	Baseline	24 Week	Change Value	*p*-Value ^(1)^		
Hair Elasticity (gf)	217.02 ± 41.27	275.16 ± 48.53	58.14 ± 40.43	<0.0001	204.91 ± 44.54	224.83 ± 40.85	19.92 ± 22.54	<0.0001	<0.0001	<0.0001
Hair Gloss (Instrumental) (GU)	3.11 ± 0.42	3.27 ± 0.39	0.16 ± 0.40	0.009	3.11 ± 0.42	3.27 ± 0.40	0.16 ± 0.37	0.005	0.996	0.996
Total Hair Count Per Unit Area (number/cm^2^)	140.40 ± 27.31	154.45 ± 24.93	14.04 ± 11.36	<0.0001	152.98 ± 25.93	157.83 ± 27.64	4.85 ± 8.60	0.0003	0.042	0.042
Hair Diameter (μm)	90.84 ± 8.58	108.34 ± 12.24	17.49 ± 6.80	<0.0001	88.31 ± 10.53	91.95 ± 9.73	3.63 ± 9.16	0.009	<0.0001	<0.0001

Values are presented as the mean ± SD; ^(1)^ analyzed by paired *t*-test between baseline and 24 weeks within each group; ^(2)^ analyzed by independent *t*-test for change value between the groups; ^(3)^ analyzed by ANCOVA (adjusted on height, weight).

**Table 7:** Hair assessment (visual inspection).

		GP Group (*n* = 47)					Placebo Group (*n* = 47)					*p*-Value ^(1)^	Adj, *p*-Value ^(2)^
		Baseline	8 Week	16 Week	24 Week	*p*-Value ^(1)^	Baseline	8 Week	16 Week	24 Week	*p*-Value ^(1)^		
Hair Gloss (Visual Inspection) (score)		1.53 ± 0.55	1.62 ± 0.61	2.30 ± 0.69	2.66 ± 0.73	<0.0001	1.55 ± 0.65	1.70 ± 0.72	2.32 ± 0.63	2.79 ± 0.69	<0.0001	0.641	0.707
Hair Distribution (score)	Crown (90°)	0.26 ± 0.51	−0.11 ± 0.43	0.17 ± 0.60	0.28 ± 0.62	<0.0001	0.03 ± 0.47	−0.06 ± 0.38	0.23 ± 0.43	0.34 ± 0.48	<0.0001	0.973	0.917
	Frontal hairline (45°)	0.20 ± 0.41	0.06 ± 0.25	0.26 ± 0.44	0.40 ± 0.50	<0.0001	0.27 ± 0.45	0.04 ± 0.20	0.32 ± 0.47	0.38 ± 0.49	<0.0001	0.539	0.728

Values are presented as the mean ± SD; ^(1)^ analyzed by linear mixed model for repeated measure data; ^(2)^ analyzed by ANCOVA (adjusted on height, weight).

**Table 8:** Satisfaction score evaluation.

	GP Group (*n* = 47)	Placebo Group (*n* = 47)	Total (*n* = 94)	*p*-Value ^(1)^
Satisfaction Score (8 w)				
1	5.81 ± 2.01	5.98 ± 2.15	5.89 ± 2.07	0.693
2	5.55 ± 2.11	5.68 ± 2.40	5.62 ± 2.25	0.785
3	6.28 ± 1.86	6.38 ± 1.95	6.33 ± 1.90	0.787
4	4.77 ± 2.22	5.38 ± 2.11	5.07 ± 2.18	0.171
5	6.91 ± 1.69	6.79 ± 2.02	6.85 ± 1.85	0.741
6	6.57 ± 1.90	6.47 ± 2.15	6.52 ± 2.01	0.800
7	6.72 ± 1.78	6.28 ± 2.09	6.50 ± 1.94	0.268
Satisfaction Score (16 w)				
1	4.91 ± 2.09	5.77 ± 1.99	5.34 ± 2.08	0.046 *
2	5.02 ± 1.92	5.06 ± 2.08	5.04 ± 1.99	0.918
3	5.45 ± 1.86	6.11 ± 1.91	5.78 ± 1.91	0.094
4	4.43 ± 2.04	5.04 ± 2.27	4.73 ± 2.17	0.170
5	5.40 ± 1.81	6.21 ± 1.81	5.81 ± 1.84	0.033*
6	5.32 ± 1.95	5.85 ± 1.78	5.59 ± 1.87	0.170
7	5.55 ± 1.94	5.83 ± 1.85	5.69 ± 1.89	0.481
Satisfaction Score (24 w)				
1	4.72 ± 1.87	5.51 ± 1.94	5.12 ± 1.94	0.049 *
2	4.66 ± 1.82	5.11 ± 2.16	4.88 ± 2.00	0.281
3	5.49 ± 2.23	5.68 ± 1.95	5.59 ± 2.08	0.658
4	4.32 ± 2.12	5.06 ± 2.07	4.69 ± 2.12	0.088
5	5.66 ± 2.04	6.26 ± 2.12	5.96 ± 2.09	0.168
6	5.45 ± 2.13	5.94 ± 2.10	5.69 ± 2.12	0.265
7	5.43 ± 2.04	5.70 ± 2.17	5.56 ± 2.10	0.526

Values are presented as the mean ± SD; ^(1)^ analyzed by independent *t*-test for change value between the groups; * *p* < 0.05.

**Table 9:** Glomerular filtration rate (GFR).

	GP Group (*n* = 47)				Placebo Group (*n* = 47)				*p*-Value ^(2)^	Adj, *p*-Value ^(3)^
	Baseline	24 Week	Change Value	*p*-Value ^(1)^	Baseline	24 Week	Change Value	*p*-Value ^(1)^		
e-GFR [60 ≤ mL/min/1.73 m^2^]	107.77 ± 10.88	106.55 ± 9.51	−1.21 ± 5.97	0.171	110.49 ± 10.65	109.09 ± 11.12	−1.40 ± 6.94	0.172	0.886	0.886

Values are presented as the mean ± SD; ^(1)^ analyzed by paired *t*-test between baseline and 24 weeks within each group; ^(2)^ analyzed by independent *t*-test for change value between the groups; ^(3)^ analyzed by ANCOVA (adjusted on height, weight).

**Table 10:** Adverse event.

	GP Group (*n* = 50)	Placebo Group (*n* = 50)	Total (*n* = 100)	*p*-Value ^(1)^	Details	Severity	Causality
Adverse Events, N (%)	1 (2.00)	0 (0.00)	1 (1.00)	1.000	Rosacea	Mild	Possibly related to test product

Values are presented as the number (%); ^(1)^ analyzed by Fisher’s exact test between the groups.

**Table 11:** Vital signs and physical examination (each visit).

	GP Group (*n* = 50)					Placebo Group (*n* = 50)					*p*-Value ^(1)^
	Baseline	8 Week	16 Week	24 Week	*p*-Value ^(1)^	Baseline	8 Week	16 Week	24 Week	*p*-Value ^(1)^	
SBP (mmHg)	114.96 ± 15.24	113.64 ± 13.14	115.86 ± 10.74	115.70 ± 12.48	0.366	111.36 ± 10.92	112.76 ± 13.73	113.72 ± 13.65	111.40 ± 12.07	0.151	0.250
DBP (mmHg)	77.84 ± 9.29	77.48 ± 9.69	79.12 ± 8.45	78.30 ± 8.29	0.395	77.96 ± 8.83	77.58 ± 10.09	77.86 ± 8.38	76.60 ± 8.83	0.452	0.426
Pulse (times/min)	74.62 ± 10.58	73.02 ± 11.37	73.98 ± 10.27	72.64 ± 10.02	0.455	76.38 ± 10.44	77.36 ± 10.66	77.44 ± 12.10	75.76 ± 10.92	0.445	0.557

Values are presented as the mean ± SD; ^(1)^ analyzed by linear mixed model for repeated measure data.

**Table 12:** Hormone testing (testosterone).

	GP Group (*n* = 50)				Placebo Group (*n* = 50)				*p*-Value ^(2)^
	Baseline	24 Week	Change Value	*p*-Value ^(1)^	Baseline	24 Week	Change Value	*p*-Value ^(1)^	
Testosterone ^†^	1.39 ± 2.44	1.27 ± 2.36	−0.12 ± 0.37	0.026 *	1.10 ± 2.30	0.93 ± 2.00	−0.17 ± 0.52	0.029*	0.618

^†^ [male 20~49 Y: 2.43~8.366, ≥50 Y: 1.93~7.40 ng/mL female 20~49 Y: 0.08~0.48, ≥50 Y: 0.03~0.41 ng/mL]; values are presented as the mean ± SD; ^(1)^ analyzed by paired *t*-test between baseline and 24 weeks within each group; ^(2)^ analyzed by independent *t*-test for change value between the groups; * *p* < 0.05.

## Data Availability

Data are contained within the article.

## Supplementary Materials

The following supporting information can be downloaded at https://www.mdpi.com/article/10.3390/nu17050767/s1, Table S1: Laboratory test indicators.

## Abbreviations

The following abbreviations are used in this manuscript:

AA	Alopecia areata
AGA	Directory of open access journals
ALP	Alkaline phosphatase
ALT	Linear dichroism
AST	Aspartate aminotransferase
CK	Creatine kinase
DHT	Dihydrotestosterone
gamma-GT	Gamma-glutamyl transferase
GCP	Good clinical practice
GFR	Glomerular filtration rate
GPAQ	Global Physical Activity Questionnaire
GP	*Gynostemma pentaphyllum*
Hb	Hemoglobin
HCG	Human chorionic gonadotropin
HDL-C	High-density lipoprotein cholesterol
HPA	Hypothalamic-pituitary-adrenal
IGF-1	Insulin-like growth factor-1
IL-6	Interleukin-6
IRB	Institutional Review Board
JAK	Janus kinase
KGF	Keratinocyte growth factor
LDH	Lactate dehydrogenase
LDL-C	Low-density lipoprotein cholesterol
MET	Metabolic Equivalent Task
PLT	Platelet
PRP	Platelet-rich plasma
RBC	Red blood cell
TNF-α	Tumor necrosis factor-alpha
VEGF	Vascular endothelial growth factor
WBC	White blood cell

## References

[1] Drake L., Reyes-Hadsall S., Martinez J., Heinrich C., Huang K., Mostaghimi A. (2023). Evaluation of the Safety and Effectiveness of Nutritional Supplements for Treating Hair Loss: A Systematic Review. JAMA Dermatol..

[2] Kesika P., Sivamaruthi B.S., Thangaleela S., Bharathi M., Chaiyasut C. (2023). Role and Mechanisms of Phytochemicals in Hair Growth and Health. Pharmaceuticals.

[3] Trueb R.M. (2015). The impact of oxidative stress on hair. Int. J. Cosmet. Sci..

[4] Mai Q., Han Y., Cheng G., Ma R., Yan Z., Chen X., Yu G., Chen T., Zhang S. (2023). Innovative Strategies for Hair Regrowth and Skin Visualization. Pharmaceutics.

[5] King B., Senna M.M., Mesinkovska N.A., Lynde C., Zirwas M., Maari C., Prajapati V.H., Sapra S., Brzewski P., Osman L. (2024). Efficacy and safety of deuruxolitinib, an oral selective Janus kinase inhibitor, in adults with alopecia areata: Results from the Phase 3 randomized, controlled trial (THRIVE-AA1). J. Am. Acad. Dermatol..

[6] Wu S., Liu S., Chen J., Dai D., Liu W., Le D., Guan Q., Miao Y., Hu Z., Qu Q. (2023). Evaluation of platelet-rich plasma plus basic fibroblast growth factor combined with minoxidil in the treatment of androgenetic alopecia: A randomized controlled trial. J. Cosmet. Dermatol..

[7] Ly N.Y., Fruechte S., Hordinsky M.K., Sadick N., Arruda S., Farah R.S. (2023). Medical and procedural treatment of androgenetic alopecia—Where are we?. J. Am. Acad. Dermatol..

[8] Tsuboi R., Niiyama S., Irisawa R., Harada K., Nakazawa Y., Kishimoto J. (2020). Autologous cell-based therapy for male and female pattern hair loss using dermal sheath cup cells: A randomized placebo-controlled double-blinded dose-finding clinical study. J. Am. Acad. Dermatol..

[9] Nestor M.S., Ablon G., Gade A., Han H., Fischer D.L. (2021). Treatment options for androgenetic alopecia: Efficacy, side effects, compliance, financial considerations, and ethics. J. Cosmet. Dermatol..

[10] Wang X., Deng Y., Wang J., Qin L., Du Y., Zhang Q., Wu D., Wu X., Xie J., He Y. (2024). New natural protein tyrosine phosphatase 1B inhibitors from *Gynostemma pentaphyllum*. J. Enzym. Inhib. Med. Chem..

[11] Xie P., Luo H.T., Pei W.J., Xiao M.Y., Li F.F., Gu Y.L., Piao X.L. (2024). Saponins derived from *Gynostemma pentaphyllum* regulate triglyceride and cholesterol metabolism and the mechanisms: A review. J. Ethnopharmacol..

[12] Yu S., Yu J., Dong X., Li S., Liu A. (2020). Structural characteristics and anti-tumor/-oxidant activity in vitro of an acidic polysaccharide from *Gynostemma pentaphyllum*. Int. J. Biol. Macromol..

[13] Wang Z., Wang Z., Huang W., Suo J., Chen X., Ding K., Sun Q., Zhang H. (2020). Antioxidant and anti-inflammatory activities of an anti-diabetic polysaccharide extracted from *Gynostemma pentaphyllum* herb. Int. J. Biol. Macromol..

[14] Kovale L., Lee S., Song M., Lee J., Son H.J., Sung Y.K., Kwack M.H., Choe W., Kang I., Kim S.S. (2024). *Gynostemma pentaphyllum* Hydrodistillate and Its Major Component Damulin B Promote Hair Growth-Inducing Properties In Vivo and In Vitro via the Wnt/beta-Catenin Pathway in Dermal Papilla Cells. Nutrients.

[15] Liu X., Lv X., Ji T., Hu H., Chang L. (2024). *Gynostemma pentaphyllum* Makino extract induces hair growth and exhibits an anti-graying effect via multiple mechanisms. J. Cosmet. Dermatol..

[16] Youssef A., Al-Mahdy D.A., Sayed R.H., Choucry M.A., El-Askary H. (2022). A Comprehensive Review of Natural Alternatives for Treatment of Alopecia with an Overview of Market Products. J. Med. Food.

[17] Song M., Kim M., Hoang D.H., Kovale L.M., Lee J., Kim Y., Lee C., Hong J., Park S., Choe W. (2022). A Hydrodistillate of *Gynostemma pentaphyllum* and Damulin B Prevent Cisplatin-Induced Nephrotoxicity In Vitro and In Vivo via Regulation of AMPKalpha1 Transcription. Nutrients.

[18] Lv J., Shen X., Shen X., Zhao S., Xu R., Yan Q., Lu J., Zhu D., Zhao Y., Dong J. (2023). NPLC0393 from *Gynostemma pentaphyllum* ameliorates Alzheimer’s disease-like pathology in mice by targeting protein phosphatase magnesium-dependent 1A phosphatase. Phytother. Res..

[19] Ke J.Y., Liu Z.Y., Wang Y.H., Chen S.M., Lin J., Hu F., Wang Y.F. (2022). Gypenosides regulate autophagy through Sirt1 pathway and the anti-inflammatory mechanism of mitochondrial autophagy in systemic lupus erythematosus. Bioengineered.

[20] Liang H.Z., Lu P.X., Chu L.L., Li G., Li C.B., Chen X.J., Zhang J., Song J., Zhang T., Luo Y. (2023). Dammarane-type saponins from *Gynostemma pentaphyllum* and their anti-aging activities via up-regulating mitochondria related proteins. Phytochemistry.

[21] Ahn Y., Lee H.S., Lee S.H., Joa K.L., Lim C.Y., Ahn Y.J., Suh H.J., Park S.S., Hong K.B. (2023). Effects of gypenoside L-containing *Gynostemma pentaphyllum* extract on fatigue and physical performance: A double-blind, placebo-controlled, randomized trial. Phytother. Res..

[22] Huang G., Yasir M., Zheng Y., Khan I. (2022). Prebiotic properties of jiaogulan in the context of gut microbiome. Food Sci. Nutr..

[23] Choi E.K., Won Y.H., Kim S.Y., Noh S.O., Park S.H., Jung S.J., Lee C.K., Hwang B.Y., Lee M.K., Ha K.C. (2019). Supplementation with extract of *Gynostemma pentaphyllum* leaves reduces anxiety in healthy subjects with chronic psychological stress: A randomized, double-blind, placebo-controlled clinical trial. Phytomedicine.

[24] Choi J.Y., Boo M.Y., Boo Y.C. (2024). Can Plant Extracts Help Prevent Hair Loss or Promote Hair Growth? A Review Comparing Their Therapeutic Efficacies, Phytochemical Components, and Modulatory Targets. Molecules.

[25] Cho Y.H., Lee S.Y., Jeong D.W., Choi E.J., Kim Y.J., Lee J.G., Yi Y.H., Cha H.S. (2014). Effect of pumpkin seed oil on hair growth in men with androgenetic alopecia: A randomized, double-blind, placebo-controlled trial. Evid. Based Complement. Altern. Med..

[26] Ham S., Lee Y.I., Kim I.A., Suk J., Jung I., Jeong J.M., Lee J.H. (2023). Efficacy and safety of persimmon leaf formulated with green tea and sophora fruit extracts (BLH308) on hair growth: A randomized, double-blind, placebo-controlled clinical trial. Skin. Res. Technol..

[27] Ortega-Quijano D., Jimenez-Cauhe J., Fernandez-Nieto D., Saceda-Corralo D., Vano-Galvan S. (2021). Comment on “Low dose oral minoxidil for treating alopecia: A 3-year North American retrospective case series”: Adding further evidence about side effects. J. Am. Acad. Dermatol..

[28] Sudeep H.V., Rashmi S., Jestin T.V., Richards A., Gouthamchandra K., Shyamprasad K. (2023). Oral and Topical Administration of a Standardized Saw Palmetto Oil Reduces Hair Fall and Improves the Hair Growth in Androgenetic Alopecia Subjects—A 16-Week Randomized, Placebo-Controlled Study. Clin. Cosmet. Investig. Dermatol..

[29] Tanuphol N., Waranuch N., Wisuitiprot V., Wisuitiprot W., Insumrong K., Temkitthawon P., Suphrom N., Jampachaisri K., Girard C., Ingkaninan K. (2024). Effectiveness and Safety of Hair Growth Formulation Containing *Tectona grandis* L.f (Teak) Leaf Extract: A Randomized, Double-Blind, Placebo-Controlled Study on Males with Androgenic Alopecia. J. Evid. Based Integr. Med..

[30] Akhbari M., Firooz A., Rahimi R., Shirzad M., Esmaealzadeh N., Shirbeigi L. (2024). The effect of an oral product containing Amla fruit (*Phyllanthus emblica* L.) on female androgenetic alopecia: A randomized controlled trial. J. Ethnopharmacol..

