# Supplementary material for: Therapeutic Potential of Gynostemma pentaphyllum Extract for Hair Health Enhancement: A Randomized, Double-Blind, Placebo-Controlled Clinical Trial
Source: Nutrients. 2025 Feb 21;17(5):767. doi: 10.3390/nu17050767 (PMC11901961; doi:10.3390/nu17050767)
Supplement: Supplementary file 1 [file nutrients-17-00767-s001.zip › nutrients-3489159-supplementary.pdf]

Supplementary Materials:

Table S1. Laboratory Test Indicators

	**GP group (n=50)**				**Placebo group (n=50)**				p-value[2)]
	Baseline	24 week	Change value	p-value[1)]	Baseline	24 week	Change value	p-value[1)]	
Complete Blood Count (CBC)									
WBC [4.00~10.00 ×103/μl]	6.09 ± 1.25	5.71 ± 1.74	-0.39 ± 1.41	0.057	5.71 ± 1.50	5.54 ± 1.37	-0.17 ± 1.19	0.315	0.408
RBC [male: 4.10 ~ 5.60 ×106/μl, famale: 3.70 ~ 4.70 ×106/μl]	4.38 ± 0.44	4.40 ± 0.44	0.02 ± 0.24	0.657	4.37 ± 0.40	4.39 ± 0.39	0.02 ± 0.23	0.521	0.912
Hemoglobin [male: 13.0~17.0 g/dl famale: 11.0~15.0 g/dl]	13.14 ± 1.34	13.27 ± 1.42	0.13 ± 0.83	0.276	13.43 ± 1.24	13.68 ± 1.19	0.24 ± 0.68	0.015	0.463
Hematocrit [male: 39.0~51.0%, famale: 33.0~45.0 %]	38.64 ± 3.68	38.93 ± 3.81	0.29 ± 2.20	0.356	39.34 ± 3.29	39.86 ± 3.12	0.52 ± 1.89	0.057	0.573
Platelet [150~370 ×103/μl]	282.94 ± 69.03	270.16 ± 64.76	-12.78 ± 25.98	0.001	275.14 ± 60.06	266.32 ± 53.68	-8.82 ± 34.02	0.073	0.515
Blood Biochemistry									
AST [male: 0~40 U/L famale: 0~32 U/L]	18.40 ± 4.20	20.70 ± 10.82	2.30 ± 10.16	0.116	18.10 ± 5.84	19.48 ± 6.09	1.38 ± 4.83	0.049	0.565
ALT [male: 0~41 U/L famale: 0~33 U/L]	15.94 ± 9.59	19.16 ± 16.34	3.22 ± 13.69	0.103	14.22 ± 7.08	16.48 ± 9.40	2.26 ± 7.60	0.041	0.666
gamma-GT [male: 10~71 U/L famale: 6~42 U/L]	18.70 ± 13.09	22.16 ± 14.71	3.46 ± 6.06	0.0002	17.38 ± 12.42	25.78 ± 25.41	8.40 ± 18.01	0.002	0.071
ALP [male: 40~129 U/L famale: 35~104 U/L]	62.78 ± 17.19	67.18 ± 19.69	4.40 ± 7.77	0.0002	60.94 ± 18.58	63.22 ± 18.31	2.28 ± 10.48	0.130	0.254

BUN [6.0~20.0 mg/dL]	12.17 ± 2.82	12.35 ± 2.90	0.17 ± 2.58	0.635	11.59 ± 3.17	11.49 ± 3.40	-0.10 ± 2.83	0.800	0.612
Creatinine [male: 0.70~1.20 mg/dL famale: 0.50~0.90 mg/dL]	0.70 ± 0.13	0.72 ± 0.13	0.01 ± 0.07	0.179	0.67 ± 0.13	0.68 ± 0.14	0.01 ± 0.07	0.166	0.964
Albumin [3.5~5.2 g/dL]	4.59 ± 0.20	4.65 ± 0.21	0.05 ± 0.18	0.039	4.60 ± 0.21	4.71 ± 0.21	0.11 ± 0.21	0.001	0.145
Total protein [6.6~8.7 g/dL]	7.24 ± 0.29	7.47 ± 0.32	0.23 ± 0.31	<.0001	7.31 ± 0.33	7.55 ± 0.38	0.23 ± 0.31	<.0001	0.897
Total bilirubin [0.00~1.20 mg/dL]	0.56 ± 0.29	0.53 ± 0.24	-0.02 ± 0.26	0.511	0.53 ± 0.26	0.63 ± 0.24	0.11 ± 0.27	0.007	0.016*
Glucose [70~99 mg/dL]	92.02 ± 6.78	95.90 ± 6.86	3.88 ± 6.14	<.0001	92.34 ± 7.23	92.64 ± 7.92	0.30 ± 8.67	0.808	0.019*
LDH [male: 135~225 U/L, famale: 135~214 U/L]	157.66 ± 29.14	160.72 ± 32.10	3.06 ± 17.75	0.229	156.64 ± 28.29	153.32 ± 26.45	-3.32 ± 21.45	0.279	0.108
Total cholesterol [0~199 mg/dL]	197.22 ± 31.03	202.46 ± 31.15	5.24 ± 26.24	0.164	197.74 ± 31.71	206.46 ± 43.82	8.72 ± 26.22	0.023	0.509
Triglyceride [0~149 mg/dL]	98.32 ± 53.05	98.52 ± 51.60	0.20 ± 43.61	0.974	101.32 ± 86.89	92.06 ± 51.11	-9.26 ± 92.50	0.482	0.515
HDL-C [40≤ mg/dL]	67.16 ± 14.70	69.30 ± 17.45	2.14 ± 8.35	0.076	69.18 ± 16.29	76.16 ± 18.41	6.98 ± 11.36	<.0001	0.017*
LDL-C [0~129 mg/dL]	121.76 ± 31.18	127.62 ± 31.89	5.86 ± 24.08	0.092	120.00 ± 28.64	126.62 ± 39.76	6.62 ± 25.16	0.069	0.878
CK [male: 0~189 U/L, famale: 0~169 U/L]	108.62 ± 74.45	113.30 ± 73.52	4.68 ± 59.70	0.582	85.90 ± 44.52	93.62 ± 54.13	7.72 ± 49.75	0.278	0.783
Urinalysis									
Specific gravity [1.003~1.030]	1.02 ± 0.01	1.02 ± 0.01	0.00 ± 0.01	0.505	1.02 ± 0.01	1.02 ± 0.01	0.00 ± 0.01	0.919	0.596
pH [4.5~7.5]	6.10 ± 0.78	6.16 ± 0.83	0.18 ± 1.27	0.678	6.27 ± 0.83	6.16 ± 0.90	-0.11 ± 1.03	0.452	0.212

Values are presented as mean ± SD

1) Analyzed by paired t-test between baseline and 24 weeks within each group

2) Analyzed by independent t-test for change value between the groups

*p<0.05
